# A Possible Smart Metering System Evolution for Rural and Remote Areas Employing Unmanned Aerial Vehicles and Internet of Things in Smart Grids

**DOI:** 10.3390/s21051627

**Published:** 2021-02-26

**Authors:** Giovanni Battista Gaggero, Mario Marchese, Aya Moheddine, Fabio Patrone

**Affiliations:** DITEN Department, University of Genoa, 16145 Genoa, Italy; giovanni.gaggero@edu.unige.it (G.B.G.); aya.moheddine@edu.unige.it (A.M.); f.patrone@edu.unige.it (F.P.)

**Keywords:** Smart Grid, Smart Metering, Internet of Things, Low Power Wide Area Network, UAV

## Abstract

The way of generating and distributing energy throughout the electrical grid to all users is evolving. The concept of Smart Grid (SG) took place to enhance the management of the electrical grid infrastructure and its functionalities from the traditional system to an improved one. To measure the energy consumption of the users is one of these functionalities that, in some countries, has already evolved from a periodical manual consumption reading to a more frequent and automatic one, leading to the concept of Smart Metering (SM). Technology improvement could be applied to the SM systems to allow, on one hand, a more efficient way to collect the energy consumption data of each user, and, on the other hand, a better distribution of the available energy through the infrastructure. Widespread communication solutions based on existing telecommunication infrastructures instead of using ad-hoc ones can be exploited for this purpose. In this paper, we recall the basic elements and the evolution of the SM network architecture focusing on how it could further improve in the near future. We report the main technologies and protocols which can be exploited for the data exchange throughout the infrastructure and the pros and cons of each solution. Finally, we propose an innovative solution as a possible evolution of the SM system. This solution is based on a set of Internet of Things (IoT) communication technologies called Low Power Wide Area Network (LPWAN) which could be employed to improve the performance of the currently used technologies and provide additional functionalities. We also propose the employment of Unmanned Aerial Vehicles (UAVs) to periodically collect energy consumption data, with evident advantages especially if employed in rural and remote areas. We show some preliminary performance results which allow assessing the feasibility of the proposed approach.

## 1. Introduction

From its birth up to nowadays, energy generation, transmission, and distribution infrastructure evolved through many steps involving many changes and improvements. Power generation is changing paradigm, from a completely centralized structure to a distributed one. The traditional electric grid structure is unable to meet new requirements such as the need of more efficient transmission means and automated fault and risk analysis, and challenges related to the integration of renewable resources [1].

The Smart Grid (SG) concept was developed to meet the aforementioned requirements and challenges. The evolution of the electrical grid towards a SG involves many aspects and requires many changes to both the current network architecture and the control functions. The main idea was to employ information and communication technologies to the electrical grid in order to improve the efficiency, sustainability and reliability [2].

The Smart Metering (SM) concept is considered as a key enabling technology for SG [3]. Smart Meters (SMes) allow not only to measure user energy consumption and forward this information more frequently in a more automatic and precise way, but also to exchange data by a two-way communication between users and utility, leading to benefits for both consumers and providers. For example, users can better manage their activities depending on the energy price, while providers can benefit from a lower management cost thanks to remote reading, configuration, disconnection/re-connection, diagnostics, outage identification, resolution of service problems, and load control [4].

Current situation is not homogeneous in all countries due to different technological levels and to the economical choices made by each Distribution System Operator (DSO). There are a lot of open challenges still to solve to make these foreseen enhancements applicable in a real scenario. Some of them are related to the chosen communication architecture that should allow a proper data exchange in different environments (urban, suburban, and rural) guaranteeing the key requirements of availability, scalability, and reliability. However, there could not be a unique solution due to the advantages and disadvantages of each possible communication protocol and to the different features of each different environment.

In this paper, we give an overview of SG and SM environments specifying the evolution of SM systems. We describe the communication solutions that can be used in SG to enhance the efficiency of the grid. We investigate the possible employment of the Internet of Things (IoT) solutions in the SG environment focusing on the SM system and we discuss a possible envisioned evolution of the SM infrastructure based on IoT protocols. In this context, we propose a novel IoT-based approach. The main idea is to propose the employment of IoT communication technologies in the SM scenario focusing on the achievable improvements compared with the current employed technologies. We evaluate the possible employment of Unmanned Aerial Vehicles (UAVs) in order to extend the application range to rural and remote areas where the use of other communication technologies could not be feasible or extremely expensive due to the lack of a proper telecommunication infrastructure. Preliminary results are reported, including real measures taken in the field, in order to show the feasibility of the proposed solution.

The paper is structured as follows. A brief introduction of the SG concept is provided in Section 2. Section 3 introduces the SM system, describing the evolution of the metering infrastructure up to nowadays, the advantages of new paradigms, and the offered functionalities. Section 4 describes possible choices in terms of communication protocols which could be employed in the different portions of the SM systems. Section 5 highlights the possible role of IoT in the future SG focusing on the SM system and its requirements. Section 6 present our IoT-based network architecture for SM system highlighting the role of UAVs to extend the coverage of IoT protocols. Conclusions are drawn in Section 7.

## 2. Smart Grids Overview

SGs include many enhancements regarding the effectiveness, efficiency, and reliability of the traditional networks by integrating communication technologies and digital computation, and are characterized by bi-directional data flows allowing the development of automated and intelligent energy systems [4].

**Figure 1:**
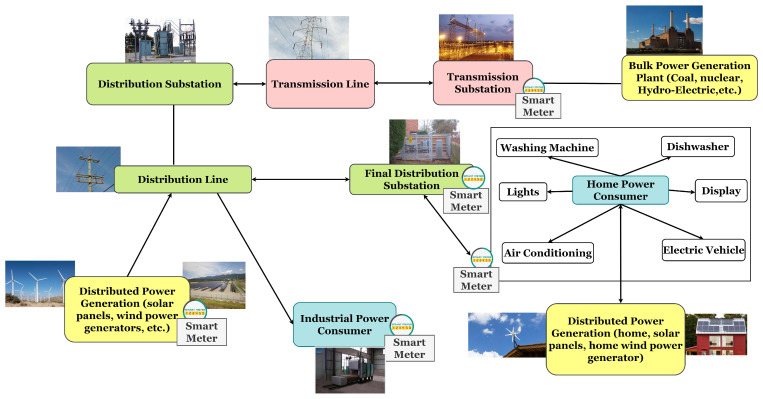
Smart Grid Electricity Flows.

SG architecture, as any other power grid, is typically divided into four subsystems: Generation, Transmission, Distribution, and Consumption, but, in a SG, power production may be also distributed. Electricity flows of a SG are shown in Figure 1.

**Figure 2:**
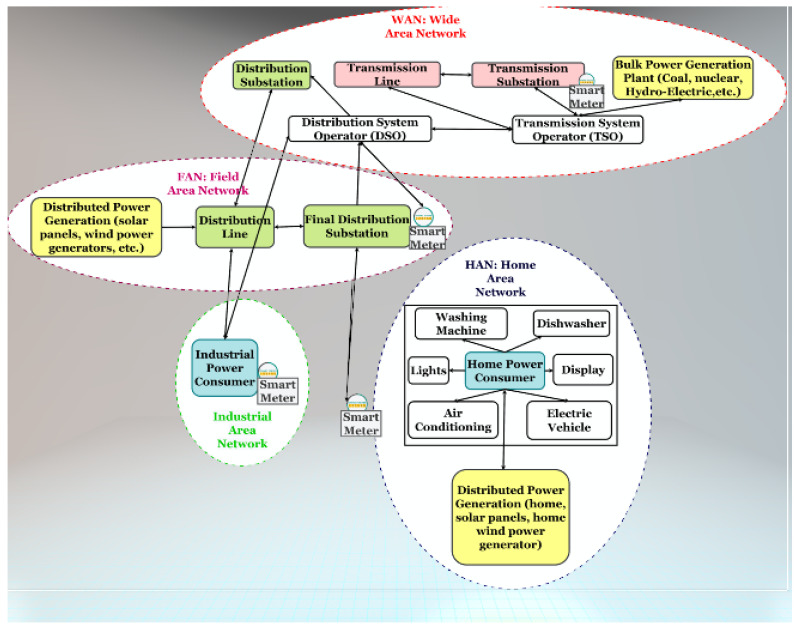
Digital information and control data flows.

Digital information and control data flows are shown in Figure 2. Data networks may be structured in three main portions [5]:

- *Home Area Network (HAN)*: the HAN is the home portion of the SG data network. It is composed of the different devices linked to the energy grid located in houses, such as power consuming televisions and washing machines, but also electric vehicles, and renewable energy resources. It connects residential devices to SMes that will allow consumers to have access to useful information, such as their energy consumption data and the current electricity price thanks to a bidirectional communication between SMes and the DSO database.
- *Field Area Network (FAN)*: the FAN is the data network for power distribution areas and includes distribution automation and control devices communicating over telecommunications networks. FAN can convey control data coming from mobile devices, feeders, reclosers, voltage regulators, capacitor banks, as well as SMes.
- *Wide Area Network (WAN)*: the WAN represents the communication backbone between substations and electric utilities. It spans over substations, DSO, and Transmission System Operator (TSO). It is the long-distance data network through which all management, control, and data storage functions are connected [2].

Except for HANs, whose border is clear, the border between FANs and WAN is quite elastic. The division proposed in Figure 2 is a possible interpretation.

The smart grid paradigm is still evolving. Many papers propose the exploitation of synergies among various forms of energy productions as a great opportunity for system improvement. These improvements can be achieved through transformation, conversion, and storage of various forms of energy in centralized units, called energy hubs, and combining transportation of different energy carriers over longer distances in single transmission devices, called energy interconnectors [6]. Another interesting concept is represented by the Smart Energy Community that can be defined as a set of energy utilities (private, public, or mixed) located in a specific area in which end-users (citizens, companies, Public Administrations, etc.) satisfy their energy needs by adopting a cooperative approach. This approach is based on the use of distributed energy generation solutions by promoting the use of renewable sources and intelligent management of energy flows in order to obtain benefits in terms of costs, sustainability, and safety. Smart Energy Communities aim to address the criticalities of the energy market and improve the resilience of the overall electrical grid [7]. However, further work is needed not only from a technical viewpoint but also in terms of legislation and social acceptability.

## 3. Smart Metering System

### 3.1. Evolution of Metering Systems: From Traditional to Smart

**Figure 3:**
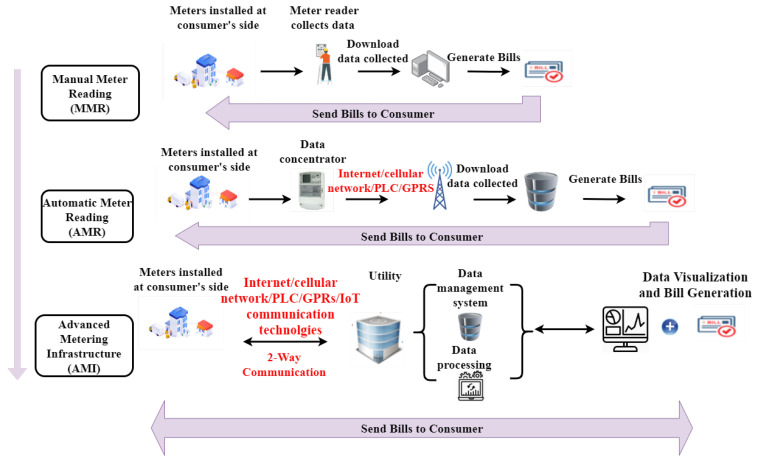
Evolution of metering systems from traditional MMR to Smart AMI.

The metering system has undergone many changes and improvements in the history from the very beginning in 1870 up to now. The metering infrastructure evolved in several steps, as shown in Figure 3:

1. *Manual Meter Reading (MMR)*: the very first metering system based on manual data collection carried on periodically (typically every 6 months or 1 year) by a person reading the shown data on mechanical meters.
2. *Automated Meter Reading (AMR)*: data measured by electronic automated meters are transferred over wired or wireless communications to the utility through intermediate nodes called data concentrators. Their aim is, on one hand, to collect data from multiple meters before sending them to the utility, and, on the other hand, to act as a relay between the two different communication technologies employed in the meters-concentrator and concentrator-utility links. Meters exploit energy cables to send data to the concentrator by employing a technique called Power Line Communication (PLC), described in detail in Section 4, which cannot guarantee end-to-end communications over long distances. Data concentrators “translate” the received data format in order to let them be forwarded through means such as cellular networks, or, if wired, optical fibers.
3. *Advanced Meter Infrastructure (AMI)*: it is characterized by the automatic transfer, process, management, and storage of user data. Differently from the previous cases, all of them based on one-way communication, AMI infrastructure enables two-way end-to-end communications and allows automatic and bidirectional metering and billing, appliance monitoring and control, and detection and diagnosis of system faults (outage). Other functionalities could be offered by this paradigm and its guaranteed bidirectional communications, such as demand-side management, detection of electricity theft, enhancement of system security, load management, emission control, and development of smart urban cities [8].

Despite the clear innovation trend, energy infrastructure upgrade is not uniform in different countries. Consequently, worldwide situation regarding the employment of SMes is not homogeneous. Even in countries with high technological development, DSOs are taking care of this upgrade carefully and prudentially for many reasons.

Italy and Sweden were the first countries in Europe to deploy SMes [9], even if nowadays other countries are planning to gradually adapt their infrastructure to the new paradigm. Numerous projects have the aim to study an efficient way to perform SMes roll-out [9]. More than 200 million smart electric meters are expected to be installed in Europe by 2023 [10], even more if we consider the employment of SMes also in other supply systems such as gas and water.

### 3.2. Traditional vs. Smart Meters

Traditional meters have no ability to store the consumption readings on a timely basis and the readers must be physically present to record the data. This is not a very efficient way, especially if these meters are installed in remote and hard to reach areas. These meters cannot detect any electric outage. Therefore, DSOs are not able to properly react to power interruptions and connections and disconnections have to be made manually. Smart meters can store consumption data on a timely basis and transmit them automatically to the DSO. They are equipped with two interfaces: one for power reading and one for data communication. DSOs can detect outages and perform quick remote reactions including connection and disconnection procedures [11].

Implementing SMes has many advantages reported in Table 1. There are also some disadvantages mainly related to the high cost of installing such meters on the premises side, the difficulty of ensuring consumer data privacy and security, and the process of switching and upgrading infrastructures from legacy to smart systems.

### 3.3. Functionalities of Smart Meters

SMes have been initially used for AMR, i.e., to read automatically the energy consumption of each user and transmit it to the DSO. The possibility to read the energy consumption many times in a day (usually every 15 min) has enabled the implementation of time-based pricing that, allowing users to save money by modifying their energy consumption profile during the day, has been the first attempt to influence the demand behaviour. The strong and fast evolution of the electrical systems suggests to carry on the implementation of new monitoring and control capabilities into SMes.

#### 3.3.1. Monitoring Capabilities

The metering capabilities of SMes can be expanded well beyond the simple energy consumption metering. Information can be generated, sent, and exploited by the DSO to manage the distribution grid so to get:

- Failure and outage notification: a fundamental issue for distribution systems is to quickly detect faults and activate protective devices. AMR-based outage management relies on the polling of SMes. Moreover, information from SMes can be used also to detect the locations of faults [12].
- Power quality monitoring: the increasing number of non-linear loads connected to the grid remarks the importance of power quality monitoring in the distribution grid, also acting at large scale [13]. Many standards have been defined to address this functionality [14] that can be implemented in a SMe [15].
- Energy theft detection: energy theft has always been a problem in distribution grids. It can be faced with new force thanks to the spread of AMI. Many approaches have been proposed in the literature based on the exploitation of AMI [16].
- Energy consumption forecast: the huge quantity of customer data can be used to obtain a better energy consumption forecast which is an important information that DSOs can exploit to manage the grid. Many innovative approaches based on machine learning have been presented, such as in [17].

#### 3.3.2. Control Capabilities

The key problem in managing an electrical infrastructure is balancing demand and energy production. Due to the intrinsic uncertainty of Renewable Energy Sources (RES) production, the DSO has to expand its control capabilities in order to improve the performance and resiliency of the grid. This action can be done in two ways: either controlling the Distributed Energy Resources (DER) power injection or modifying the load consumption profile. In the first case, when a private user installs a DER source, such as a solar panel, the DSO may require to remotely send commands to the electronic converter in order to either reduce or turn off the power injection. Controlling DER power injection may be a fundamental tool to avoid over-voltages in areas with a high penetration of DER. The second possibility is to influence the power consumption of a customer to match power demand and supply. The action stands in the framework of demand side management and demand response, which may offer a broad range of potential benefits to system operations by allowing the interaction and responsiveness of the users [18]. Demand response programs can be categorized by: the control mechanism (centralized or distributed); the offered motivations (price-based or incentive-based), and the decision variable (task scheduling-based or energy management-based) [19]. For example, a smart home could buy/sell energy from/to the grid taking into account time-varying non-linear price variations, as proposed in [20]. From a communication viewpoint, different strategies involve different requirements. For example, demand response is a strong time-critical application.

Another fundamental issue is represented by the spread of Electric Vehicles (EVs). Uncontrolled charging strategies of EVs may result in severe problems for the grid. Controlled strategies can be categorized into two main families: smart and bidirectional charging [21,22]. In the first case, power flows every time from the grid to the vehicles. A coordination strategy between vehicles is required to help avoid load peaking. In the second case, power can flow in a bidirectional way depending on the needs of the grid and on the constraints imposed by the customers by exploiting the storage capabilities of the EVs’ battery. If an EV implementing the bidirectional charging strategy is connected within the customer domain, the SMe can enable the communication with a remote control center. Bidirectional charging can be further classified in Vehicle to Grid (V2G), Vehicle to Vehicle (V2V) and Vehicle to Home (V2H) systems.

## 4. Communication Protocols and Standards

The main aim of SG is to integrate advanced communication technologies within the power grids to collect generated data, extract useful ones and enhance the efficiency of the grid [23]. Hence, the successful implementation of a SM system depends on the used communication technology [24] that must have low cost, assure proper transmission range and bandwidth, provide security features and low power consumption [25]. The chosen communication technology differs according to the three network portions of the SG mentioned in Section 2. This differentiation is based on network coverage, connection type, and application to be deployed [26].

Communication solutions can be categorized in *wired* and *wireless* technologies.

Wired technologies are robust to interference but require the installation of fixed infrastructure. Wireless technologies have lower installation costs and are more suitable to be deployed in rural and remote areas. They are versatile, provides reliable and scalable solutions through the possible addition of new nodes [27]. Low PowerWide Area Networks (LPWANs) are a category of wireless communication protocols developed for emerging IoT applications but also applicable in the SM systems.

**Figure 4:**
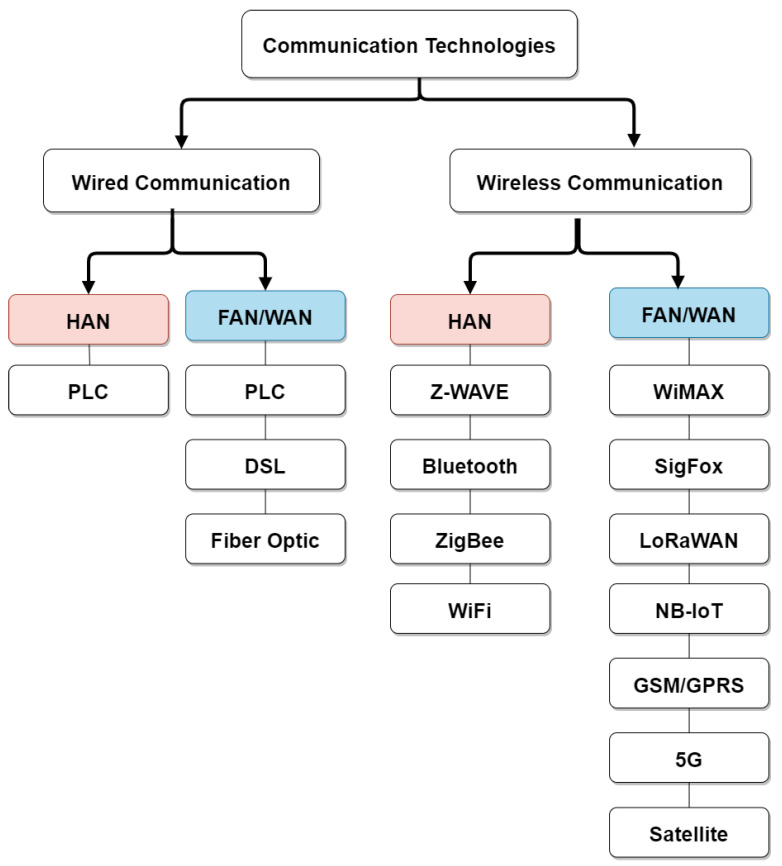
Wired and wireless communication technologies used in HAN, FAN, and WAN.

Figure 4 summarizes the solutions belonging to the two mentioned categories highlighting the SG portions where their employment is more suitable. Detailed descriptions are reported below.

### 4.1. Wired Communication Technologies

Main wired technologies that can be employed are:

- *PLC*: it is the first communication technology used in SM systems taking advantage of the presence of power lines cables. It is classified into two categories: Narrowband PLC (NB-PLC) and Broadband PLC (BB-PLC). Both of them have a coverage in the order of kilometers. Data rates can raise up to 500 kbps for NB-PLC and to hundreds of Mbps for BB-PLC. This communication technology is widely used in SM systems due to the already present and deployed medium [24]. However, bandwidth restrictions in urban areas limit: PLC applications that require higher bandwidth; the number of devices connected to power lines; and the distance separating the transmitter and receiver, which affects the signal quality [2,28].
- *Digital Subscriber Line (DSL)*: it is an high speed digital data transmission technology that uses the wires of the telephony network. It can cover a distance up to 5 km with data rates ranging between 1.5 and 12 Mbps. Despite these advantages, its maintenance cost is considered to be very high and its efficiency and performance decrease when the distance increases [24].
- *Optical Fiber*: it is one of the most feasible communication means in WANs due to its noise immunity and the high achievable data rate. It offers good performance over long distances up to 60 km even if it requires high installation cost and it is hard to upgrade. Different forms of optic fiber are available depending on the application and the quality of services the network delivers such as Passive Optical Network (PON), Wavelength Division Multiplexing (WDM), and Synchronous Optical Networking (SONET) [2,24,29,30].

Table 2 represents a comparison between the wired communication technologies with respect to their data rates, coverage, and latency.

### 4.2. Wireless Communication Technologies

Main wireless technologies that can be employed are:

- *Z-Wave*: it has been designed especially for remote control applications in residential environments. This technology offers reliable, low data rate, and low bandwidth solution for HAN applications that require short range communication [32].
- *ZigBee*: it is a micro-power wireless technology based on IEEE 802.15.4 standard, a high-efficient standard for peer-to-peer and personal area networks. It provides an efficient and cost effective solution for home automation, healthcare, electric, and many more applications. ZigBee offers low data rates, low power consumption, low delay, and secure communications for HAN SG applications [33].
- *Bluetooth*: it is a wireless communication technology designed for short range data exchange, it offers fast data exchange and low power consumption. It is suitable for the communication between smart home devices and SMes within the HAN.
- *WiFi*: it is based on the IEEE 802.11 standard and offers medium distance coverage and medium/high data rates. It is suitable for applications within the HAN tier with low interference ratio/environments.
- *WiMax*: it is based on the IEEE 802.16 standard and has been designed for Wireless Metropolitan Area Network (WMAN). This wireless technology provides low latency and secure communication. It is characterized by its low operation costs and scalability. Due to its bandwidth and coverage range, it is suitable for data exchange within the FAN supporting SG applications such as the detection and restoration of outages and wireless automatic meter reading and monitoring [32].
- *Cellular Networks (GSM, GPRS, LTE, 5G)*: such technologies are suitable for the communication between the subsystem elements and the data management system/utility in any SM applications. It offers low deployment costs since the network already exits (or it will be deployed in the near future for the 5G) making it easier for the utilities to focus on services and applications [9,32].
- *Narrowband IoT (NB-IoT)*: a narrowband LPWAN technology that is compatible with LTE, GSM and GPRS technologies. It offers low energy consumption and wider signal coverage solution [34,35]. This technology is suitable for the FAN portion due to the coverage it offers supporting AMI applications.
- *LoRaWAN*: it is an LPWAN IoT protocol that offers long range coverage that can reach 15 km in rural areas, low power consumption, low cost, and low data rates. It has been initially designed to build urban platforms for IoT, new it is also a good candidate for SG applications [36] since this technology offers low cost deployment and can be efficiently used for the communication between devices (equipped with LoRa interface) and SMes in the HAN and in the FAN due to its long range coverage.
- *SigFox*: it is a proprietary IoT technology suitable for low noise level environments offering long range, low power, and low data rates. It is considered an ideal solution for smart cities and SG applications [37]. Since it offers wide coverage, it is suitable to be used in the FAN portion.
- *Satellite communications*: satellites can provide worldwide connectivity, particularly useful in remote and rural areas without any other kind of telecommunication infrastructure and to high-speed moving platforms, such as planes and ships, making it suitable for WAN applications. However, this type of communication may be sensitive to weather conditions which highly affect the data transmission [30].

Table 3 represents a comparison between the wireless communication technologies with respect to their data rates, coverage, and latency.

## 5. Internet of Things in Smart Grids

### 5.1. IoT Applications in Smart Grid

Focusing on communication, different networking technologies provide communication services for the different SG applications [40]. IoT is considered as an effective technology for different SG applications. IoT communication solutions can, on one hand, allow data gathering, processing, and exchange among different physical elements or components of the SG [2,41,42,43,44,45], while, on the other hand, improve the SG abilities, such as warning, disaster recovery, and reliability. It is considered to be a reliable mean of data transmission whether wired or wireless through different SG parts. IoT can be deployed to monitor power generation, energy storage, energy consumption, transmission lines and substations, and can be installed on the customer side SMe for consumption measurement and energy management purposes [46].

To establish the IoT integration within SG, several IoT architectures have been proposed which can be classified into two different architectures: 3-layer and 4-layer architectures. The 3-layer IoT architecture composed of perception, network, and application layers has been adopted in [47,48]. Perception layer corresponds to the physical objects, sensors of different kinds, RFID tags, cameras, etc. The main role of these elements is to collect data from the surrounding and, thanks to the related communication module, send these data to the network layer. The network layer represents the bridge connecting the perception to the application layer. It is composed of different wired (PLC, DSL, optical fiber) and wireless (WiFi, ZigBee, LoRaWAN, 4G) communication protocols. It is responsible for routing the gathered data to be processed at the application layer. The final layer is used for data processing and monitoring of these devices in real-time. On the other hand, the 4-layer IoT architecture for SG has been considered in [49].

**Figure 5:**
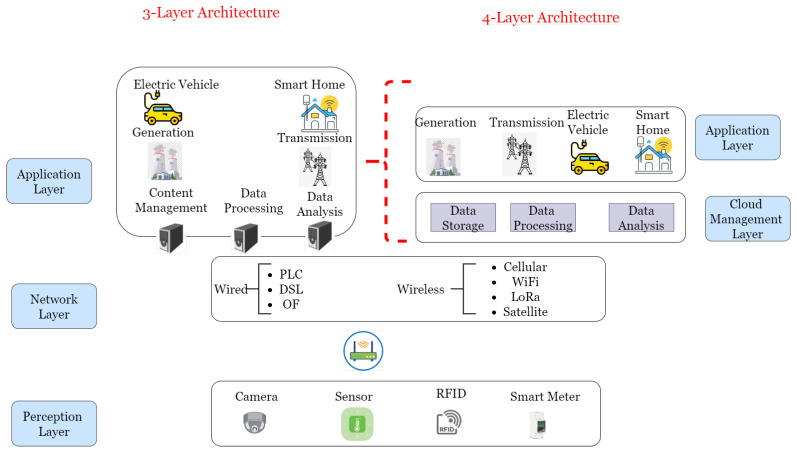
IoT-based SG architecture [48,49].

Figure 5 shows the two possible IoT-based SG architecture.

The newly added layer, called “Cloud Management Layer”, is placed between the network and application ones. It is responsible for data storage and information retrieval while the application layer is in charge of offering the different SG services such as demand-response management, energy management and dynamic pricing. Authors in [50] followed the 4-layer IoT architecture but with a twist. The authors proposed a new model composed of terminal, field network, remote communication and master station system layers. SMes and devices compose the terminal layer; the field network layer corresponds to both wired and wireless communication technologies; the remote communication layer also includes the communication technologies but the ones that offer wide area connectivity as 4G cellular networks; finally, the master station system layer corresponds to the control systems of the different SG parts (generation, transmission, distribution).

A new architecture for IoT-based SG supported by edge computing is presented in [51]. The authors proposed a 5-layer architecture composed of *devices layer*, *network layer*, *data layer*, *application layer*, and *cloud computing layer*.

In order to allow SG data transmissions throughout the Internet, the use of widespread telecommunication architectures is also under investigation, completely or partially replacing the ad-hoc defined communication system of former “energy” networks [5]. The communication solutions defined for IoT use cases seem suitable for this purpose also considering the similarities between the features of the data traffic flows in SG and in some typical IoT scenarios.

Several studies investigated the possible employment of these IoT-thought solutions in the SG scenario: the LPWAN category [52]. Thanks to its guaranteed low power consumption, achievable long transmission range, and ensured security and data protection through encryption, the solutions of this category seem of special interest in this context. A detailed description of the architecture of the Chinese SG when integrating IoT communication technologies is given in [53], also presenting some applications where IoT solutions can be of great benefit, such as for online monitoring of power transmission lines, smart patrolling, smart home services, and electric vehicle management. An overview of SGs and of the different applications and services where IoT is integrated into SG is provided in [46]. The integrated architecture is also presented concerning the necessary requirements of privacy, security, and reliability. LoRaWAN is used as a highly reliable communication technology for a SG installed in a rural area due to the coverage limitations of the cellular network in [54]. Supporting the Green Bali Provenance Program, authors in [55] evaluate the communication solutions belonging to the LPWAN category to deploy two-way communication SMes replacing pre and postpaid digital ones. The performance results suggest the adoption of LoRaWAN, thanks to its low implementation cost, low power consumption, device availability, long distance coverage, and frequency allocation. Authors in [56] also evaluates the LoRa protocol performance deploying one gateway and several SMes distributed over a 9-floors building. The results show that a single gateway is enough to receive data from thousands of SMes. An application that uses EnergyHive [57] as energy platform is presented in [58]. Such a system consists of about 300 properties, each equipped with a SMe connected through WiFi and a tablet to display the energy consumption in real-time. SMes are used to provide automatic energy management through real-time consumption monitoring ability and report generation. IoT technologies use the information from SMes to adjust the energy consumption within the building. The achievable performance of LoRaWAN in rural SGs has been investigated in [59] by deploying the SG over an area of 4000 square kilometers and offering electric power to about 200 consumers. The obtained results show the importance of sending small data payload packets to avoid long latency. LoRaWAN is used as a communication protocol to transmit the data generated by SMes in [60]. The aim is to study the Quality of Service (QoS) that the LoRaWAN solution can provide in a crowded city like Paris. The performance analysis shows good results in terms of guaranteed QoS but the network capacity decreases when both uplink and downlink communications are considered. The role of IoT communication is extended to overcome the problem of voltage regulation when deploying distributed energy resources. The authors in [61] presented an IoT model for voltage regulation by using sensors and actuators installed on micro-grids and 5G cellular communication to transfer the information about the grid status.

### 5.2. IoT Applications in Smart Metering Systems Operating in the Field

IoT in the domain of SM applications has been emerging as a research field. Some studies were conducted by using IoT-based communication technologies in the field of power management. An IoT architecture has been adopted in [62] for the implementation process of a model predictive control of Heating Ventilation and Air Condition (HVAC) systems in smart buildings. The implemented system uses different sensors and actuators connected to a gateway by using ZWave and ZigBee communication protocols. The system is connected to a database via Message Queuing Telemetry Transport (MQTT) to allow the users to remotely control it. a management system to control the increasing energy demand-response due to the growing spread of IoT-enabled smart homes is proposed in [63]. An improvement of the IoT-enabled smart home sustainability is guaranteed by following a strategy which controls and manages the power consumption during peak times, reduces the power cost, and increases users comfort. An Energy Management System (EMS) to monitor domestic devices energy consumption and utilization is presented in [64]. The authors adopt a stochastic-based scheme to save energy in smart grid applications, which can be extended to cover all IoT components in a smart city. An Intelligent Smart Energy Management System (ISEMS) is introduced in [65] to handle the increasing energy demands in SG environments and to accurately predict energy consumptions by using machine learning prediction models.

Different applications in SM systems based on the LoRaWAN solution have been deployed in the field, not necessarily linked to SGs:

- Water Grid Transformation-Birdz [66]: a smart water metering network with LoRaWAN-based sensors has been deployed in France. The integration allows the utility to collect data more efficiently and control operations to reduce costs. This implementation provides a flexible deployment of sensors, such as water leak detectors and water quality probes, which can send and receive messages through a two-way communication network. Significant benefits have been obtained such as identification and faster repair of about 1200 water leaks in the distribution network. It allowed saving 1 million cubic meters of water on an annual basis.
- Nationwide Smart Metering [67]: a unique energy control system based on LoRaWAN devices has been developed by a Czech communication company in cooperation with a technological start-up. This system collects real-time data from electrical meters installed in houses and industries and allows users to check their usage by using a smartphone application.
- Creating Energy Efficient Buildings [68]: a company in China developed a system to monitor their utility buildings through LoRaWAN-based devices. Building owners and managers can monitor usages and tune consumption in order to reduce waste.
- Energy Management and Smart Lighting-OrionM2M [69]: a lighting system based on LoRaWAN networks to access the cloud has been developed in Kazakhstan. This connectivity enables reliable transmission of data, reduces the cost, and creates a more efficient lighting system.

### 5.3. The Importance of Security, Privacy, Timeliness, and Data Accuracy

Increasing the number of SMes, the amount of data traversing the system, and the SG size, the concerns about network security and data privacy also increase. AMI can be considered as a cyber-physical system, which means that attacks against the communication media affect the behaviour of the physical system. The risk level strongly depends on the functions implemented in the SMes. Actually, while compromising billing measures leads to economic damages to the DSO, attacks against remote control capabilities can have a huge impact on the core service provision of the electrical grid. Confidentiality, integrity, and availability are, in order of importance, the key requirements in Information Technology (IT) environments. In Operation Technology (OT) systems, the importance of these requirements is usually reversed. OT systems include the management of processes that, if not executed correctly, pose a significant risk to the health and safety of human lives, damage to the environment, and serious financial issues, such as production losses. Availability is essential in this context and, for this motivation, redundancy is an integral part of the system, also concerning the involved communication systems. Redundancy is required where failures cannot be tolerated and in critical applications such as substation automation. At the same time, availability requires that the employed security mechanism, including but not limited to cryptographic systems, shall not degrade the maintainability, operability, and accessibility in case of emergency. Integrity is another key point: generated, transmitted, displayed, and stored data should be kept genuine and intact without unauthorized intervention. AMI systems involve both the security of the electrical grid and the privacy of the customers. For this reason, communications between customers and DSO is a very critical issue.

Data gathered by SMes is of high importance for different parties. One of the customer’s main interest is to avoid unauthorized entities from accessing their data and expose their energy habits. Confidentiality is an issue. If SMes and utilities do not exchange data in a secure way, malicious and unauthorized people can get access to this information. This would result in a severe violation of the privacy of the customers. This information can be utilized to track the habits of the inhabitants of the house, with the related risks for the peoples lives. To read confidential data, alter control commands, and ban authorized operators to access the system are just a few examples of dangerous activities that can be performed. Security does not involve only SMes but also all the components of the SM architecture. These components may face different threats that affect their performance and security [70]. From the consumer side, SMes have to protect the generated energy consumption data and avoid unauthorized parties from accessing these important and critical information. These data have to be encrypted and any changes or tampers must be prevented and denied.

The network between the SMes and the utility is exposed to different kinds of attacks, especially considering if it is located in the external environment, widespread throughout the covered area, and with an end-to-end connection that can be based on different communication technologies, both wired or wireless. Protection mechanisms have to be implemented to react to possible cable cuts, infrastructure damages, and radio interferences.

Table 4 summarizes some of the main possible security threats against SM systems.

Timeliness is the last key requirement: it explicitly expresses the time-criticality of systems. It includes both the responsiveness aspect of a system, e.g., a command from controller to actuator should be executed in real-time, and the timeliness of any related data being delivered in its designated time period. In a nutshell, all data should be processed at the right time.

Another important aspect is to guarantee IoT data recovery. Data losses can occur due to problems or attacks to wireless communications, as mentioned in Table 4. Such losses affect the entire system, so a recovery mechanism to retrieve these data is strongly needed. A model to increase the prediction accuracy to recover lost data by using deep learning algorithms is proposed in [72]. Other studies adopt different machine learning algorithms to complete the missing data in datasets [73,74].

### 5.4. Communication System Requirements

Consequently to what expressed in the previous section, both energy consumers and DSOs have certain requirements that need to be guaranteed. In SGs, the considered communication solutions need to be properly evaluated and chosen in order to be sure they can fulfil all network requirements. Specific aspects have to be considered in this analysis concerning the communication system [5,28,31,75]:

- Availability: the system has to be always active and available to all users. The coverage of the communication technology has to be taken into account to allow the SG to cover all users, especially the ones located in rural and remote areas.
- Scalability: the system should have the ability to scale up when new substations and meters are added and to continue to guarantee requirements and services to each user.
- Reliability and Resilience: the system has to be robust and assure end-to-end data delivery in a secure way. Redundancy mechanisms should also be considered to keep the SG operative in case of local faults or malicious attacks.
- QoS: the system has to guarantee a proper level of accuracy of the collected data to both users and providers. The SG has to always assure the defined QoS to each user even increasing user density per square kilometer and the overall number of devices connected to the SG, conditions that could lead to congestion situations and degradation of the communication performance (information loss, outage or delay), which would also affect the pricing process [76].
- Data amount and network rates: the amount of data that has to traverse each portion of the SG needs to be considered in the system design. Communication technologies and protocols have to be consequently selected also considering the maximum amount of information they can send over time units.
- Latency: some SG applications are delay sensitive (see the reference to timeliness above). The communication solutions for data exchange among the involved entities need to consider and fulfil time constraints.

Different tasks may have different requirements. For example, traditional tasks, such as metering for billing purposes, are not time-critical and a possible failure does not impact the safety of the overall electric system, but for many applications is not so. Table 5 summarizes the performance requirements concerning bandwidth, reliability, and latency for different functionalities in the distribution grid.

## 6. Possible Smart Metering System Evolution

### 6.1. Our Envisioned System

Among the different communication solutions discussed in Section 4, there could not be a “best” solution. The most suitable one may depend on many factors, including the already present infrastructure, the geography of the areas, and the functionalities that the DSO wants to implement.

Our vision of possible future evolution of the SM system is based on the employment of IoT protocols and in particular the LPWAN ones. This solution is mainly based on the equipment of LPWAN solutions on the SMes. In this way, information exchange from and to SMes can be achieved both in urban and rural scenarios at low implementation cost.

**Figure 6:**
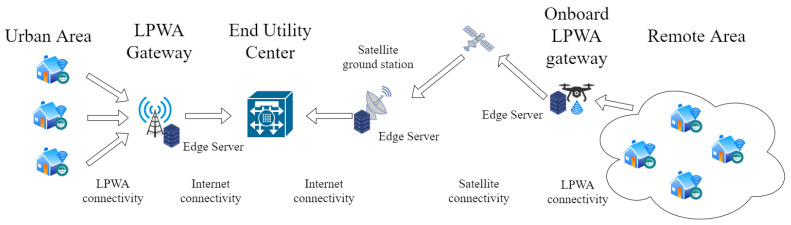
Proposed SM system architecture integrating IoT communication and MEC paradigm.

The overall SM system can so be composed of two main possible scenarios depicted in Figure 6:

- Scenario A: SM in Urban Areas
  Fixed LPWAN solution gateways are deployed in specific locations aims to minimize their number while covering all the present SMes. These gateways are directly connected with the SMes receiving/forwarding data from/to them. These nodes can also be equipped with high-computational and storage capabilities, exploiting the Mobile Edge Computing (MEC) paradigm, in order to allow them performing data analysis and processing at the edge of the network. In this way, the system can avoid forwarding the raw data to the end utility and allow the implementation of the mentioned additional functionalities closer to the user and the distribution portion of the network reducing the latencies. Another interface will allow processed data to be forwarded to the central End Utility nodes through the Internet exploiting more traditional communications solutions such as Fiber Optics or Cellular Network.
- Scenario B: SM in Remote Areas
  Many areas are rural and remote areas with low population density. In such areas, the deployment of fixed LPWAN gateways would be cost-inefficient. Besides, considering they could be out of coverage of all other traditional communication technologies, the links between the gateways and the End Utility centers could not be easy to establish. To solve this issue, we propose the use of UAVs equipped with LPWAN gateways. In this way, UAVs can be easily deployed in the areas to cover, establish bidirectional communications with the SMes, and proceed with the data exchange, acting as moving LPWAN gateways. Satellite communication solutions can offer the missing link between UAVs and End Utility centers thanks to their envisioned future worldwide coverage. Another viable solution could be to allow the UAVs to operate as “data mule”, i.e., collect the data while are flying above the rural area, store onboard the data and then proceed to forward them only when they come back to their starting point. This second solution is less expensive than the first one even if it cannot guarantee direct end-to-end connectivity between SMes and End Utility centers. The inclusion of data server at the edge is also possible in this second scenario even if the most feasible locations could be the UAVs or, considering the limited available resource onboard the UAVs and maximum payload weight limitations, the satellite ground stations.

In urban areas, LPWAN can be a practical, low cost, low energy consumption, and easy to deploy solution. It does not require a deep modification of the SM system and it would exploit the Internet infrastructure to forward data to the End Utility Centers instead of using an ad-hoc infrastructure. However, high SMes density and the high interference typical of an urban scenario are the main drawbacks that could lead to different solutions employing more traditional communications means, more expansive but more able to guarantee the performance and security requirements. Anyway, the concept of exploiting the MEC paradigm at the gateway nodes and the Internet infrastructure is still valid.

In rural and remote areas, the proposed approach is very practical and cost effective. UAV flight path and frequency may depend on the structure of the area to cover, on the locations of the SMes, and on the different needs. Energy measurements as frequent as in urban areas may not be feasible but it will be anyway a considerable improvement compared to the MMR case, currently adopted in almost all rural areas. Moreover, in the data mule solution, UAVs can be easily moved through different environments, but different moving objects can be also employed for this purpose, such as cars, taxis, and busses, as has been already proposed in other data communication initiatives [77,78,79,80].

The employment of the MEC concept in both urban and rural areas is the turning point to enable all the mentioned functionalities and make the future SG able to manage the higher amount of generated information traversing the all infrastructure and satisfy the user higher performance requirements offload part of the computation load from the End Utility Centers to the Edge Servers. This allows reducing network load and bandwidth utilization, enhancing service performance, and decreasing the overall system delay.

The exploitation of Internet infrastructure is a further improvement. In this way, there is no need anymore of an ad-hoc infrastructure with the related periodic maintenance and upgrade costs. There are already Internet application protocols able to guarantee the needed security requirements and solutions to guarantee enough bandwidth to satisfy both users and DSO performance requirements.

Moreover, the applicability of the proposed solution may not be limited only to SMes. Other entities of the SM system, belonging to all the portions of the SG infrastructure, can benefit from the employment of IoT communication protocols and the presence of flying UAVs to send and receive data. Control messages from transmission lines, alerts from distribution substations, security and condition checks from multiple distributed power generation locations are just a few examples of the data that DSOs and TSOs could easily and efficiently collect thanks to the proposed approach.

### 6.2. UAV-IoT Gateway Prototype

**Figure 7:**
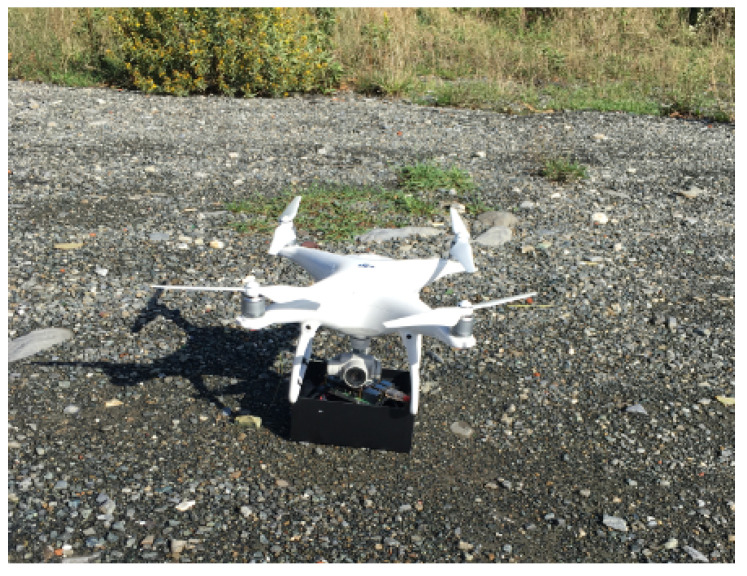
UAV equipped with an IoT LoRaWAN gateway.

In order to verify if a UAV can be suitable for the envisioned purpose, both from a logical and a technological viewpoint, we are developing a prototype based on a quadcopter drone equipped with a LoRaWAN gateway [81]. This flying gateway is based on a Raspberry Pi 3 B+ equipped with the LoRa shield RAK2245 Pi-Hat, powered by a battery pack, and attached to a DJI Phantom UAV, as illustrated in Figure 7.

**Figure 8:**
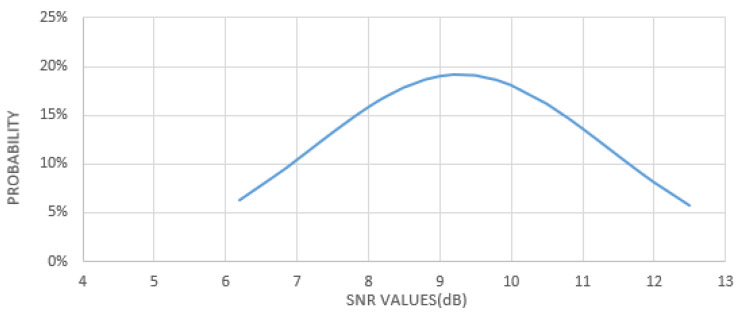
Density function of the SNR values measured during the test.

Some preliminary tests have been performed where the sensors act as smart meters and the gateway installed onboard the drone carried out some flights testing the system’s feasibility. In detail, two IoT devices have been placed in an open area at an approximate distance of 100 m from each other. The UAV flew above the area at an altitude of 20 m for approximately 20 min following a random path. During that time, each sensor kept sending data at a packet rate of one packet every 30 s instead of one packet for the entire test duration, in order to emulate the presence of a higher number of data sources. Figure 8 shows the density function of the Signal-to-Noise Ratio (SNR) measured by the gateway.

**Figure 9:**
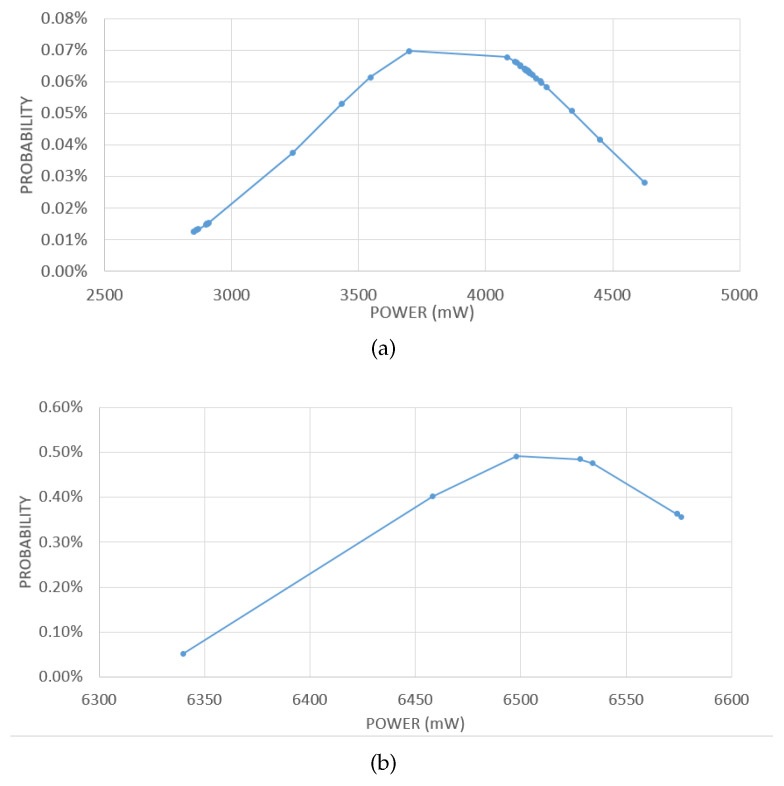
Density functions of the power consumed by the IoT gateway during the test while waiting to transmit (**a**) and transmitting data (**b**).

Taking into account the importance of energy consumption in such applications, a special sensor has been attached to the battery pack used to power the IoT gateway in order to monitor and record the power and current consumption of the gateway when receiving and transmitting data from sensors. Figure 9 shows the density function of the power consumed by the IoT gateway to stay active and receive/transmit data, i.e., while waiting to transmit and transmitting data.

The shown collected SNR values reflect the typical values achievable in this kind of communication links, proving the feasibility of the proposed solution in terms of achievable performance.

The shown measured power consumption values highlight a reasonable power consumption of the IoT gateway. Even a typical lightweight battery with low maximum capacity is enough to power the gateway for all the maximum flight time of typical UAVs (usually a few hours).

Additional tests have been performed and the obtained results have been included and described in one of the previous articles of the same authors [82].

Even if it has been currently tested in simpler conditions than the ones of a possible SM system real case scenario, it already proved its feasibility and advantages also applied to the SM system use case.

## 7. Conclusions

New technologies and communication protocols are leading to an evolution of the energy grid towards the Smart Grid paradigm. The SM system could be enhanced by the integration of these solutions, improving its current capabilities and including additional functionalities. Both users and utilities will deeply benefit from this infrastructure upgrade, especially thanks to a bidirectional end-to-end communication between smart meters and utilities that can allow a better management of the available resources and an operational cost reduction. Different communication solutions are suitable to be integrated and employed in SGs in order to avoid using ad-hoc protocols and infrastructure, but additional concerns must be taken into account.

We considered the possible useful employment of IoT protocols within the SG focusing on the SM system. Possible network architecture improvements have been proposed highlighting the needed requirements, the main issues that have to be considered in the infrastructure design, and the pros and cons of each identified communication solutions, in order to shed the lights on how the energy grid could further evolve in the future.

We propose a possible network architecture based on the employment of IoT protocols of the Low Power Wide Area Network category and of a UAV-based IoT gateway to directly collect data from SMes. This configuration can be especially useful in remote and rural areas where the employment of solutions based on a fixed infrastructure can be hindered by several disadvantages such as the geographical structure of these locations and the required economical investments. Some information related to an under-development prototype and a reference to some preliminary results has been included.

We focus our investigation on IoT technologies and their possible employment within the considered scenario because we think they could be a viable choice to improve the data exchange and provide the current SM systems with additional functionalities. However, different approaches could be considered and investigated with the same aim and lead to solutions that could be compared with the solution proposed in this paper.

Future activities could be dedicated to further developing and testing on the proposed solution also considering more realistic scenarios including real node distribution and data generation.

## Figures and Tables

**Table 1:** Advantages of SM system for both utilities and consumers.

Utilities	Consumers
accurate meter reading and improved billing	accurate costs and no more estimation-based bills
decrease in staff costs due to the automatic meter reading and decrease in energy waste	energy consumption monitoring allowing the reduction in bills costs
increase in the reliability and quality of the services provided through the real-time monitoring of the metering system	energy theft detection by monitoring the high increase in bill costs
fraud detection through the generation of behaviour profile of the consumer	intelligent decision making by using information provided by meters
asset management and maintenance enhancement	fault management due to near real-time monitoring

**Table 2:** Comparison between the different wired communication technologies [26,28,31].

Technology	Standard	Data Rate	Coverage	Latency	Application	Pros	Cons
*PLC*	Narrowband NB-PLC	10–500 Kbps	up to 3 km	High	HAN, FAN	Cost effective	Network topology and number of devices may affect the signal quality
	Broadband BB-PLC	14–200 Mbps	up to 200 m					Low
*DSL*	ADSL	1–8 Mbps	up to 5 km	High	FAN	Low installation cost due to existing infrastructure	Low reliability, regular maintenance and distance dependent
	HDSL	2 Mbps	up to 3.6 km				
	VDSL	15–100 Mbps	up to 1.5 km				
*Optical fiber*	AON (IEEE 802.3ah)	100 Mbps	up to 10 km	Low	FAN/WAN	High bandwidth capacity	High cost
	BPON (ITU-TG.983)	1.2 Gbps	10–20 km				
	GPON (ITU-TG.984)	155 Mbps–2.448 Gbps	up to 60 km				
	EPON (IEEE 802.3ah)	1.25 Gbps	10–20 km				
	SONET/SDH	10 Gbps	up to 100 km				
	WDM	40 Gbps	up to 100 km				

**Table 3:** Comparison between different wireless communication technologies [2,26,27,28,31,38,39].

Technology	Frequency	Data Rate	Coverage	Application Area	Pros	Cons
*Z-Wave*	868 MHz 908 MHz	40 kbps	30 m (indoor) 100 m (outdoor)	HAN	low power consumption	low data transmission rate
*ZigBee*	868–915 MHz	250 kbps	10–100 m	HAN	low complexity low deployment cost	low data rate short range
*Bluetooth*	2.45 GHz	721 Kbps	1–100 m	HAN	low power consumption	low data rate signal lose in noisy environments
*WiFi*	2.4, 5 GHz	54 Mbps	up to 250 m	HAN	high data rate	high power consumption
*WiMAX*	2–11 GHz 11–66 GHz	up to 70 Mbps	up to 50 km	FAN/WAN	high data rate low installation cost	shared bandwidth
*2G-GSM*/ *GPRS*	900–1800 MHz	14.4 Kbps 56–114 Kbps	3–50 km	FAN/WAN	already existing infrastructure security algorithms implemented	shared network with non-grid applications and users
*3G-UMTS*	380 MHz–2.5 GHz	384 kbps–7.2 Mbps	5–75 km	FAN/WAN	already existing infrastructure security algorithms implemented	shared network with non-grid applications and users
*4G-LTE*	700 MHz 850 MHz 900 MHz 1800 MHz 2100 MHz 2300 MHz 2600 MHz	50 Mbps UL, 100 Mbps DL	3–12 km	FAN/WAN	low latency high speed high bandwidth	high cost
*NB-IoT*	900–1800 MHz	250 Kbps (UL) 230 Kbps (DL)	35 km	FAN/WAN	ultra-low power consumption low cost and complexity	high latency
*5G*	sub 6 GHz mm-wave	up to 10 Gbps	up to 50 km	FAN/WAN	very low latency very high speed very high bandwidth	high cost
*LoRaWAN*	868–870 MHz 902–928 MHz 915–928 MHz 470–510 MHz	0.3–50 Kbps	up to 5 km (urban) up to 15 km (rural)	FAN/WAN	low power consumption low installation cost secure bidirectional communication	possible signal interference
*Satellite*	1–40 GHz	a few kbps- a few Gbps	400 km–36,000 km	WAN	wide coverage	high latency

**Table 4:** Description of possible security threats on a SM system [71].

Security Threat	Description	Possible Attack Place
*Listening*	unauthorized interception of private communications known as Eavesdropping. Such threat allows analyzing the information intercepted.	HAN, FAN, and WAN
*Modification*	unauthorized interception of data to change or cancel them. Such threat allows the modification of data collected from meters and leads to the generation of invalid demand-response commands, false connect/disconnect commands and price signal changing.	FAN and WAN
*Denial of Service*	unauthorized parties may exhaust all available resources.	FAN and WAN
*Cheating Consumer*	the ability of the consumer to attack the end utility to change or reduce the bill cost. This can be done by resetting the meter and re-program it to give invalid data.	HAN
*Packet Injection*	the injection or inoculation of incorrect packets. This may be done for different reasons such as to lower the power for some parts of the metering system, modify the billing process and generate false bills.	FAN and WAN networks

**Table 5:** Performance requirements in the distribution grid [5].

Functionalities	Bandwidth	Reliability	Latency
AMI	10–100 kbps	99.0–99.99%	2000 ms
Demand Response Management	14–100 kbps	99.0%	500 ms—a few mins
Outage management	56 kbps	99.0%	2000 ms
Distribution Automation	9.6–56 kbps	99.0–99.99%	20–200 ms
Distribution Management	9.6–100 kbps	99.0–99.99%	100 ms–2 s
Asset Management	56 kbps	99.0%	2000 ms
Meter Data Management	56 kbps	99.0%	2000 ms
Distributed Energy Resources	9.6–56 kbps	99.0–99.99%	300 ms–2 s
V2G	9.6–56 kbps	99.0–99.99%	2s—a few mins
Electrical Vehicles Charging	9.6–56 kbps	99.0–99.99%	2s—a few mins
Home Energy Management	9.6–56 kbps	99.0–99.99%	300–2000 ms

## Data Availability

Date sharing not applicable.
